# Multidimensional Evaluation of Combined Anticoagulation and Venoprotective Therapy in Deep Vein Thrombosis: A Retrospective Propensity Score-Matched Cohort Study of Clinical, Economic, and Resource Utilization Outcomes

**DOI:** 10.3390/reports8020083

**Published:** 2025-06-01

**Authors:** Nan Zhou, Teck Han Ng, Chai Nien Foo, Lloyd Ling, Yang Mooi Lim

**Affiliations:** 1 Centre for Cancer Research, M. Kandiah Faculty of Medicine and Health Sciences, Universiti Tunku Abdul Rahman, Kajang 43000, Selangor, Malaysia; zhounan@1utar.my (N.Z.); foocn@utar.edu.my (C.N.F.); 2 Department of Medicine, M. Kandiah Faculty of Medicine and Health Sciences, Universiti Tunku Abdul Rahman, Kajang 43000, Selangor, Malaysia; ngth@utar.edu.my; 3 Department of Population Medicine, M. Kandiah Faculty of Medicine and Health Sciences, Universiti Tunku Abdul Rahman, Kajang 43000, Selangor, Malaysia; 4 Lee Kong Chian Faculty of Engineering and Science, Universiti Tunku Abdul Rahman, Kajang 43000, Selangor, Malaysia; linglloyd@utar.edu.my; 5 Department of Pre-Clinical Sciences, M. Kandiah Faculty of Medicine and Health Sciences, Universiti Tunku Abdul Rahman, Kajang 43000, Selangor, Malaysia

**Keywords:** venous thrombosis [C14.907.355.830], drug therapy, combination [E02.319.267], treatment outcome [N05.715.360.775.700]

## Abstract

**Background**: Deep vein thrombosis (DVT) management remains challenging despite standard anticoagulation therapy. This study evaluated the comprehensive benefits of combining rivaroxaban with Aescuven (CAV) compared to rivaroxaban monotherapy (SAT) in DVT treatment. **Methods**: A retrospective analysis was conducted on DVT patients (2018–2023) using multi-method propensity score matching and ensemble weighting. Outcomes included improvement rate (IPR), daily improvement rate (DIR), cost-effectiveness ratio (CER), daily improvement cost (DIC), cost–LOS efficiency (CLE), and length of stay (LOS). Counterfactual analysis was implemented to estimate causal effects. **Results**: The CAV group demonstrated superior outcomes compared to SAT: IPR increased by 6.39 percentage points (95% CI: 5.61–7.39), DIC substantially reduced by 3323.38 CNY (95% CI: 2887.95–3758.81), and CLE improved by 136.97 CNY per day (95% CI: 122.31–151.64), with minimal LOS increase (0.15 days, 95% CI: 0.12–0.18). Network analysis revealed significant correlations between baseline coagulation parameters and treatment outcomes, particularly between APTT and economic benefits. **Conclusions**: The CAV regimen achieved significant clinical and economic advantages over standard monotherapy without substantially increasing resource utilization. These findings support integrating venoprotective agents into conventional anticoagulation strategies for optimized DVT management.

## 1. Introduction

Deep vein thrombosis (DVT) represents a significant cardiovascular condition characterized by thrombus formation within deep veins, predominantly affecting the lower extremities [1]. The pathogenesis follows Virchow’s triad: blood stasis, endothelial injury, and hypercoagulability. Global epidemiological data indicate an annual incidence of 1–2 cases per 1000 individuals, with higher rates observed in elderly populations and specific risk groups [2,3]. The condition demonstrates substantial morbidity, as 20–50% of untreated DVT patients develop post-thrombotic syndrome, while 2–4% progress to fatal pulmonary embolism [4]. Multiple risk factors contribute to DVT development, including prolonged immobilization, major surgery, trauma, malignancy, and inherited thrombophilia [5]. The economic burden of DVT management on healthcare systems exceeds USD 10 billion annually in developed nations, emphasizing the critical need for effective therapeutic strategies [6].

Rivaroxaban, a direct factor Xa inhibitor, has established itself as a cornerstone in DVT treatment due to its predictable pharmacokinetics and elimination of routine monitoring requirements. Clinical trials demonstrate its non-inferiority to traditional vitamin K antagonists with a favorable safety profile [7]. Aescuven, with its active ingredient aescin (some of the literatures refers to it as escin or β-escin) derived from horse chestnut seed extract, serves as a complementary therapeutic agent in DVT management through its multi-targeted pharmacological actions. It exhibits anti-inflammatory effects by suppressing leukocyte activation and adhesion molecule expression, while simultaneously enhancing vascular integrity through the stabilization of capillary endothelium and reduction of vascular permeability [8,9]. Clinical evidence has demonstrated its effectiveness in ameliorating lower extremity edema and enhancing microcirculatory function, making it a valuable adjunctive therapy in comprehensive DVT treatment [10,11].

Despite the established efficacy of rivaroxaban monotherapy, clinical challenges persist in DVT management, including residual thrombosis, post-thrombotic syndrome development, and suboptimal symptom resolution. The integration of venoprotective agents with standard anticoagulation represents a promising therapeutic strategy that may address these limitations through complementary mechanisms of action. However, comprehensive evaluations of combined regimens that simultaneously assess clinical efficacy, economic impact, and resource utilization outcomes remain scarce. This study aims to conduct a multidimensional evaluation of combined anticoagulation and venoprotective therapy (rivaroxaban plus Aescuven) compared to standard anticoagulation therapy (rivaroxaban alone) in DVT patients, utilizing advanced propensity score matching methodologies and counterfactual analysis to establish causal relationships between treatment modalities and outcomes across clinical, economic, and healthcare resource utilization domains.

## 2. Materials and Methods

### 2.1. Research Design and Data Collection Process

This retrospective study analyzes clinical data from DVT patients treated at [Institution Name Withheld for Blind Review] (2018–2023), derived from “A machine learning-based model for the management of inpatients with deep vein thrombosis” (ethics approval: SSMYY-KYPJ-2023-011). This study includes patients with primary DVT diagnosis and complete medical records, excluding cases with incomplete treatment or unavailable consultations. Data collection by vascular specialists covers patient demographics, clinical parameters at admission, and DVT symptom scores based on the 2015 Diagnostic Criteria [12] (Supplementary File Table S1). The dataset, initially used for machine learning modeling in DVT management, underwent secondary analysis to evaluate the therapeutic benefits of combined Aescuven and rivaroxaban therapy. A structured SDV process ensures data quality, with a hierarchical protocol for resolving verification issues. Due to the retrospective nature of this study, the Sun Simiao Hospital of Beijing University of Chinese Medicine waived the need of obtaining informed consent. All methods were performed in accordance with the relevant guidelines and regulations.

### 2.2. Variable Definitions

This study incorporates six key outcome variables to comprehensively evaluate treatment effectiveness and resource utilization. The improvement rate (IPR) quantifies the relative symptom reduction during hospitalization, calculated as [(Day 1 symptom score—Discharge symptom score)/Day 1 symptom score × 100%], representing the overall therapeutic effectiveness. The daily improvement rate (DIR) normalizes the IPR by length of stay (LOS), computed as IPR/LOS, reflecting the speed of symptom improvement. The cost-effectiveness ratio (CER) measures economic efficiency by dividing total hospitalization costs by absolute symptom score reduction. The daily improvement cost (DIC) represents the financial investment required for each unit of daily symptom improvement, calculated by dividing total hospitalization costs by DIR. The cost–LOS efficiency (CLE), derived from CER/LOS, evaluates resource utilization efficiency by incorporating both economic and temporal dimensions. The length of stay (LOS) represents the total hospitalization duration, serving as a fundamental indicator of healthcare resource consumption. All cost-related outcomes (CER, DIC, and CLE) are reported in Chinese Yuan (CNY), with each unit representing the amount of currency required to reduce one point in the symptom score.

### 2.3. Group Definition

This study conducted a secondary analysis of an existing clinical cohort of DVT patients. Based on the therapeutic regimens documented in the cohort, patients were stratified into two groups. The combined anticoagulation and venoprotective therapy group (CAV group) consisted of patients who received dual therapy with rivaroxaban plus Aescuven, while the standard anticoagulation therapy group (SAT group) included patients who received rivaroxaban monotherapy. This classification enables a direct comparison between standard anticoagulation and the enhanced therapeutic approach incorporating venoprotective agents.

### 2.4. Multi-Method Propensity Score Matching and Ensemble Weighting

To address potential selection bias and ensure robust comparative analysis, a multi-method matching approach was implemented when the sample size ratio between groups exceeded 2:1. This comprehensive strategy employed four distinct matching algorithms: genetic matching, Mahalanobis distance matching, nearest neighbor matching with caliper, and optimal matching with exact constraints. The matching variables encompassed demographic factors (age, gender, height, weight), clinical parameters (Wells score), and laboratory values (WBC, RBC, Hgb, PLT, HCT, PT, INR, APTT, TT, Fib, D-Dimer, FDP) obtained on the first day of admission. Each matching method generated a distinct matched dataset.

### 2.5. Ensemble Weighting Framework for Causal Effect Estimation

To enhance the robustness of causal inference, we developed a comprehensive multidimensional evaluation framework for weighting different propensity score matching methods. This framework integrates five key dimensions of methodological quality to determine the optimal contribution of each matching algorithm in the final analysis. First, covariate balance was assessed using both mean and maximum standardized mean differences (SMD) across all baseline variables, with weights inversely proportional to these metrics to prioritize methods achieving superior balance. Second, we evaluated the predictive performance of each matched dataset through five-fold cross-validation, measuring the average R^2^ across all outcome variables to capture how well the matched data preserved meaningful relationships between covariates and outcomes. Third, we conducted Rosenbaum sensitivity analysis to quantify the robustness of treatment effect estimates against potential unmeasured confounding, calculating the proportion of outcomes maintaining statistical significance at gamma threshold of 1.5. Fourth, sample size retention was considered as a secondary criterion to balance statistical power with matching quality. The final ensemble weights were calculated using a weighted formula: mean SMD balance (25%), maximum SMD balance (15%), predictive performance (25%), Rosenbaum sensitivity robustness (30%), and sample size retention (5%). This integrated approach combines the strengths of multiple matching methods while mitigating their individual limitations, resulting in more stable and reliable causal effect estimates.

### 2.6. Comprehensive Causal Effect Analysis

Counterfactual analysis was implemented to estimate the causal effects of CAV versus SAT across multiple clinical outcomes. The framework employed random forest models to predict potential outcomes under alternative treatment scenarios. For each outcome variable, separate models were trained on the treated and control groups to capture potentially heterogeneous relationships between baseline covariates and outcomes. These models generated counterfactual predictions (y_1_, y_0_) representing what would have occurred had all patients received either treatment or control conditions. Individual treatment effects (ITE) were calculated as the difference between these counterfactual predictions (y_1_-y_0_), while average treatment effects (ATE) were derived as the mean of these individual differences. Treatment effects on the treated (ATT) and controls (ATC) were computed by averaging ITEs within the respective subpopulations. To enhance robustness, a double residual approach was implemented, wherein both outcome and treatment assignment were regressed on baseline covariates, with the relationship between resulting residuals providing an alternative treatment effect estimate. Variable importance measures were extracted from the random forest models to identify key confounders influencing treatment outcomes. The analysis was performed independently across four matched datasets, with final estimates calculated as weighted averages using the previously established ensemble weights. This comprehensive counterfactual framework enabled the assessment of treatment effect heterogeneity while accounting for complex non-linear relationships between baseline characteristics and clinical outcomes.

### 2.7. Software and Code Availability

All matching analyses were performed using R version 4.2.1 (R Foundation for Statistical Computing, Vienna, Austria). The counterfactual analysis was conducted using Python version 3.11, with random forest models implemented for predicting counterfactual outcomes. To ensure reproducibility and transparency, the complete analysis code has been made publicly available at https://github.com/ZhouNan2020/ACV_RV (accessed on 25 May 2025 ).

## 3. Results

### 3.1. Cohort Characteristics and Matching

**Figure 1:**
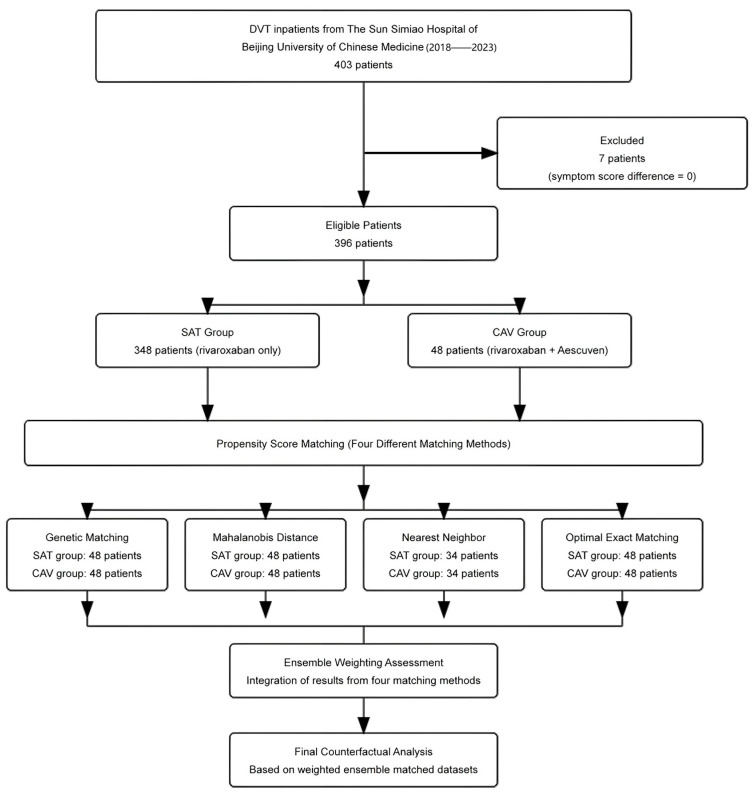
Patient flow diagram and propensity score matching strategy.

**Figure 2:**
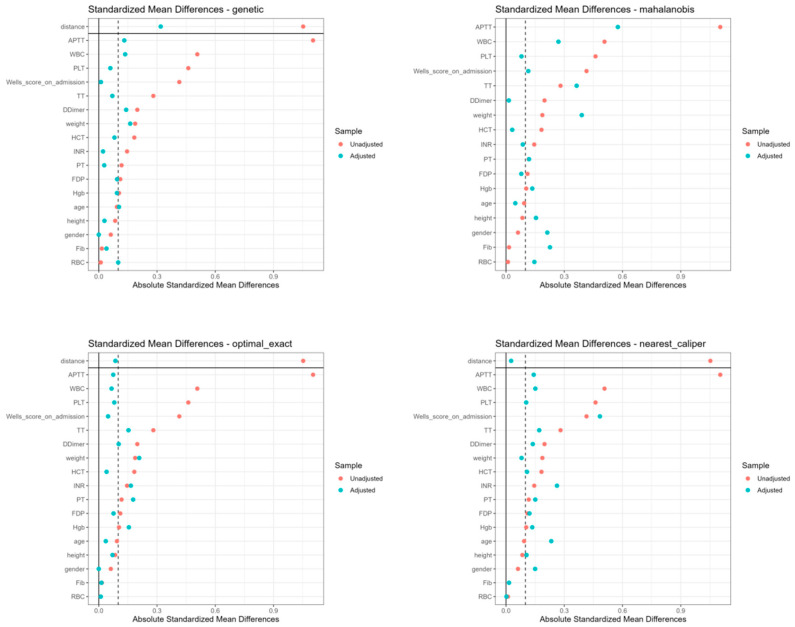
Standardized mean differences of baseline characteristics across four matching methods.

The initial cohort comprised 403 DVT inpatients from the Sun Simiao Hospital of Beijing University of Chinese Medicine (2018–2023), with 7 patients excluded due to symptom score difference of zero, which prevented calculation of cost-effectiveness ratios. Among the 396 eligible patients, significant baseline imbalances were observed between the SAT group (*n* = 348, rivaroxaban only) and CAV group (*n* = 48, rivaroxaban plus Aescuven), particularly in gender distribution (56% vs. 40% female, *p* = 0.035), white blood cell count (9.95 vs. 9.25 × 10^9^/L, *p* = 0.009), platelet count (158 vs. 146 × 10^9^/L, *p* = 0.048), APTT (33.5 vs. 28.9 s, *p* < 0.001), and TT (24.8 vs. 27.0 s, *p* = 0.003). To address these imbalances, four propensity score matching algorithms were implemented, each demonstrating distinct performance patterns in achieving covariate balance. Nearest neighbor caliper matching achieved superior balance with all standardized mean differences (SMDs) below 0.1, particularly in laboratory parameters (D-Dimer: 0.02, HCT: 0.03, RBC: 0.04) and clinical indicators (Wells score: 0.01), though it retained the fewest patients (34 in each group). Optimal exact matching showed strong performance in demographic variables (gender: 0.00, age: 0.06) and most laboratory values (PT: 0.02, INR: 0.03), while maintaining the full CAV group sample size (48 patients in each group). Genetic matching maintained acceptable balance across variables (SMDs < 0.3), with excellent performance in coagulation parameters (PT: 0.05, INR: 0.04) but larger differences in distance metrics (0.82). Mahalanobis distance matching exhibited variable performance, achieving good balance in some parameters (D-Dimer: 0.02, HCT: 0.04) while retaining larger differences in others (APTT: 0.42, PLT: 0.38, WBC: 0.35), indicating less consistent overall balance compared to other methods (Figure 1 and Figure 2; Table 1; Supplementary File Table S1).

### 3.2. Ensemble Weighting Evaluation

**Figure 3:**
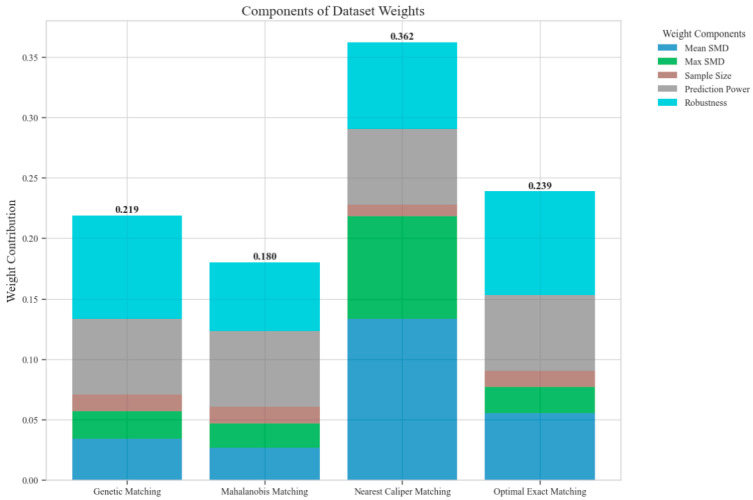
Components of dataset weights across different matching methods.

The ensemble weighting evaluation revealed distinct performance patterns across the four matching methods. Nearest caliper matching demonstrated superior overall performance with the highest composite weight (0.362), driven by exceptional covariate balance (mean SMD = 0.055) and robust predictive performance (R^2^ = 0.521) for length of stay outcomes. Optimal exact matching and genetic matching achieved moderate weights of 0.239 and 0.219, respectively, with notable strengths in robustness against unmeasured confounding. Mahalanobis distance matching received the lowest weight (0.180) due to suboptimal covariate balance (mean SMD = 0.272) and sensitivity analysis performance. The predictive performance analysis revealed heterogeneous R^2^ values across outcome variables, with particularly strong performance in LOS prediction (R^2^ ranging from −0.019 to 0.521) and moderate performance in IPR prediction (R^2^ ranging from 0.171 to 0.232). Rosenbaum bounds analysis demonstrated robust treatment effects for CLE and IPR outcomes, maintaining significance at higher gamma values, while effects on DIR and DIC showed sensitivity to unmeasured confounding at lower gamma thresholds (Figure 3).

### 3.3. Causal Effect Analysis Results

**Figure 4:**
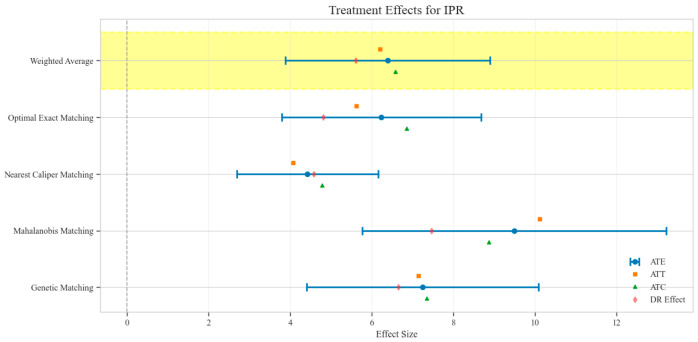
Comparison of treatment effect estimates for IPR across different matching methods (Yellow background: Ensemble weighted average across all matching methods).

**Figure 5:**
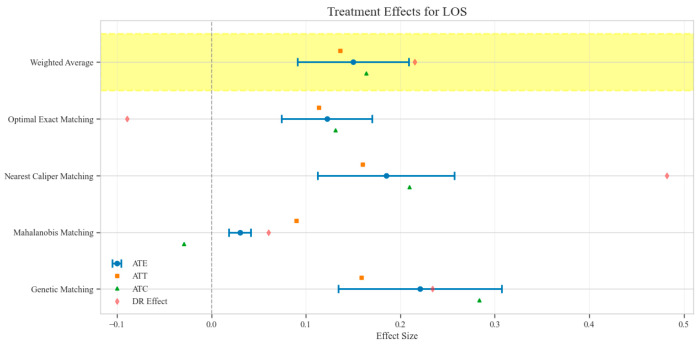
Comparison of treatment effect estimates for LOS across different matching methods (Yellow background: Ensemble weighted average across all matching methods).

**Figure 6:**
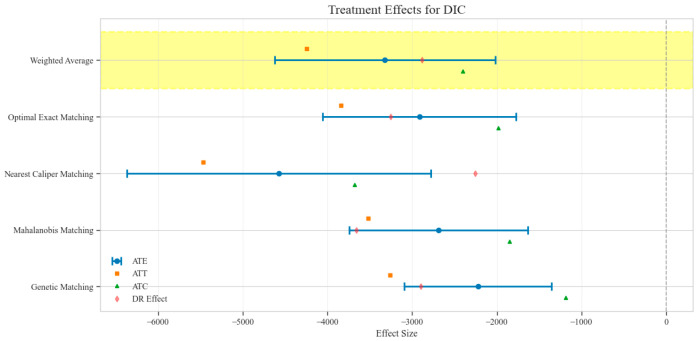
Comparison of treatment effect estimates for DIC across different matching methods (Yellow background: Ensemble weighted average across all matching methods).

**Figure 7:**
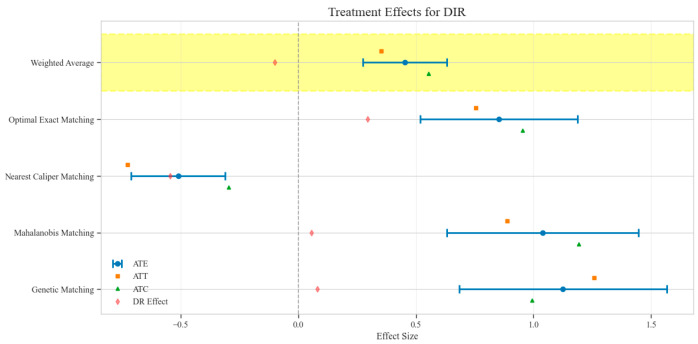
Comparison of treatment effect estimates for DIR across different matching methods (Yellow background: Ensemble weighted average across all matching methods).

**Figure 8:**
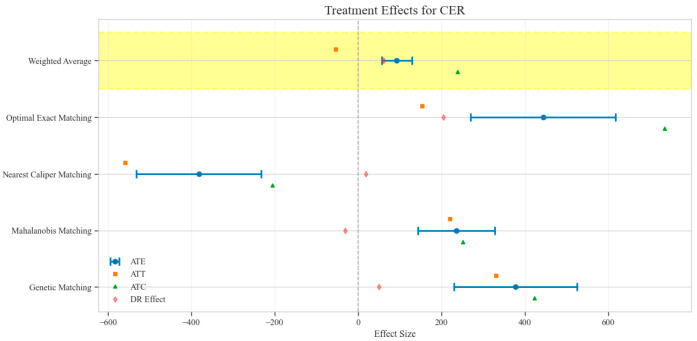
Comparison of treatment effect estimates for CER across different matching methods (Yellow background: Ensemble weighted average across all matching methods).

**Figure 9:**
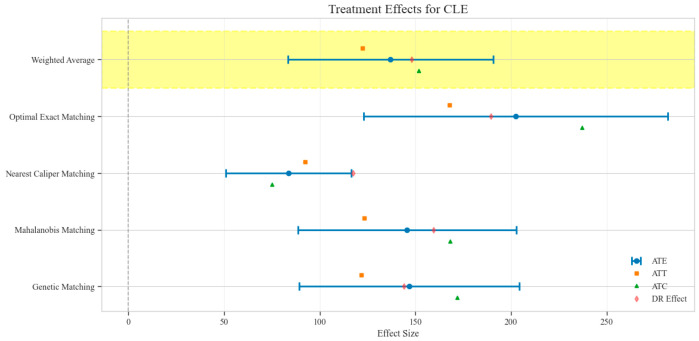
Comparison of treatment effect estimates for CLE across different matching methods (Yellow background: Ensemble weighted average across all matching methods).

The counterfactual analysis across multiple matching methodologies demonstrates consistent treatment effects of the CAV intervention compared to the SAT control group. The weighted average estimates reveal that the CAV group achieved a higher IPR with an ATE of 6.39 percentage points, while maintaining comparable LOS with only a marginal increase of 0.15 days. The economic outcomes showed pronounced advantages, with DIC substantially reduced by 3323.38 CNY, indicating lower financial expenditure required for symptomatic improvement. The DIR exhibited a positive ATE of 0.45 units, confirming faster symptom resolution in the CAV group. CER analysis revealed an ATE of 92.49 units, with the ATT showing a favorable negative value of −53.79 units, demonstrating enhanced economic efficiency in the CAV group. The CLE demonstrated a positive ATE of 136.97 units, further substantiating CAV’s advantage in resource utilization efficiency. Notably, ATT values for the CAV group consistently showed more favorable results than ATC values for most economic indicators, reinforcing the treatment’s beneficial effects on the treated population through rigorous causal inference methodology (Table 2; Figure 4, Figure 5, Figure 6, Figure 7, Figure 8 and Figure 9)

**Figure 10:**
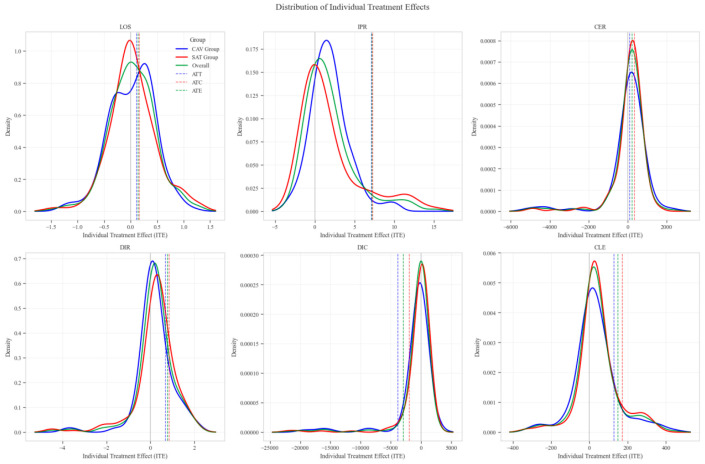
Distribution patterns of individual treatment effects across six clinical outcomes.

The distribution analysis of ITE across six clinical and economic outcomes demonstrates pronounced heterogeneity between treatment groups with consistent advantages for the CAV intervention. The density plots reveal distinctly favorable ITE distributions for the CAV group in IPR, with rightward-shifted density curves indicating superior therapeutic effectiveness compared to the SAT group. For DIR, the CAV group similarly exhibits advantageous distribution patterns, with ATT and ATE values consistently positive, demonstrating accelerated symptom improvement. Notably, the DIC distribution predominantly occupies negative territory, with CAV group density concentrated further left than the SAT group, signifying substantially reduced financial expenditure per unit of clinical improvement. The CLE distribution further corroborates CAV’s economic advantage, with rightward-shifted densities confirming enhanced resource utilization efficiency. The LOS distribution shows minimal between-group differences, though with slight reduction trends in the CAV group. The CER exhibits tightly overlapping distributions with marginally favorable positions for the CAV group. These findings conclusively establish the CAV intervention’s superior performance across therapeutic efficacy and economic efficiency dimensions at the individual patient level (Figure 10).

**Figure 11:**
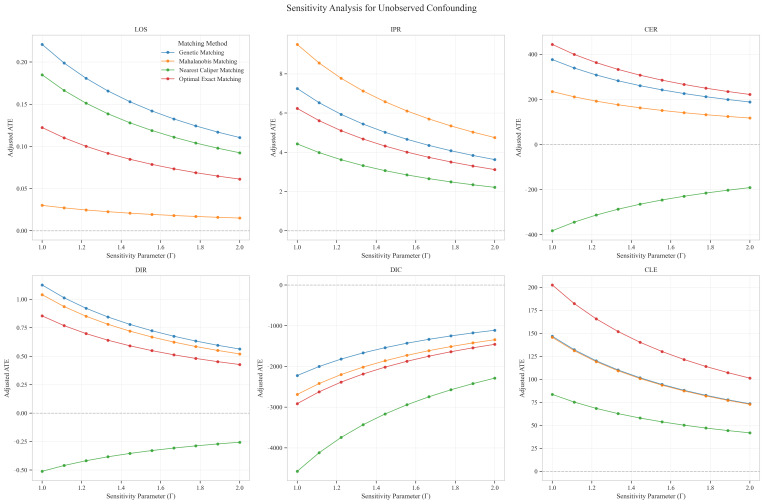
Sensitivity analysis of treatment effect estimates to unobserved confounding across six clinical outcomes.

Sensitivity analysis for unobserved confounding demonstrated varying robustness across outcome measures as the sensitivity parameter (Γ) increased from 1.0 to 2.0. For LOS, all matching methods showed positive ATE values that consistently decreased with increasing Γ, ranging from approximately 0.03–0.22 at Γ = 1.0 to 0.02–0.11 at Γ = 2.0, indicating minimal but persistent differences in hospitalization duration favoring the CAV group. IPR exhibited strong positive effects across all matching methods, with ATE values ranging from approximately 4–9 at Γ = 1.0 to 2–5 at Γ = 2.0, suggesting robust therapeutic advantages for CAV even under strong confounding scenarios. CER displayed heterogeneous patterns: three matching methods showed positive values (200–400 at Γ = 1.0, decreasing to 150–300 at Γ = 2.0), while nearest caliper matching yielded negative values (−400 at Γ = 1.0, increasing to −200 at Γ = 2.0), indicating method-dependent economic efficiency assessments. DIR showed method-dependent effects with three matching approaches yielding positive values (0.5–1.0 at Γ = 1.0, decreasing to 0.4–0.6 at Γ = 2.0), while nearest caliper matching produced negative values (−0.5 at Γ = 1.0, increasing to −0.25 at Γ = 2.0), suggesting variable speed of symptom improvement. DIC consistently displayed negative values across all methods (−2000 to −4500 at Γ = 1.0, increasing to −1500 to −2200 at Γ = 2.0), indicating substantially lower daily improvement costs in the CAV group even under strong confounding assumptions. CLE showed positive values across all methods (75–200 at Γ = 1.0, decreasing to 45–100 at Γ = 2.0), suggesting better resource utilization efficiency in the CAV group. Overall, the CAV group demonstrated consistent advantages in IPR, DIC, and CLE, while showing method-dependent results for DIR and CER, with minimal differences in LOS (Figure 11).

**Figure 12:**
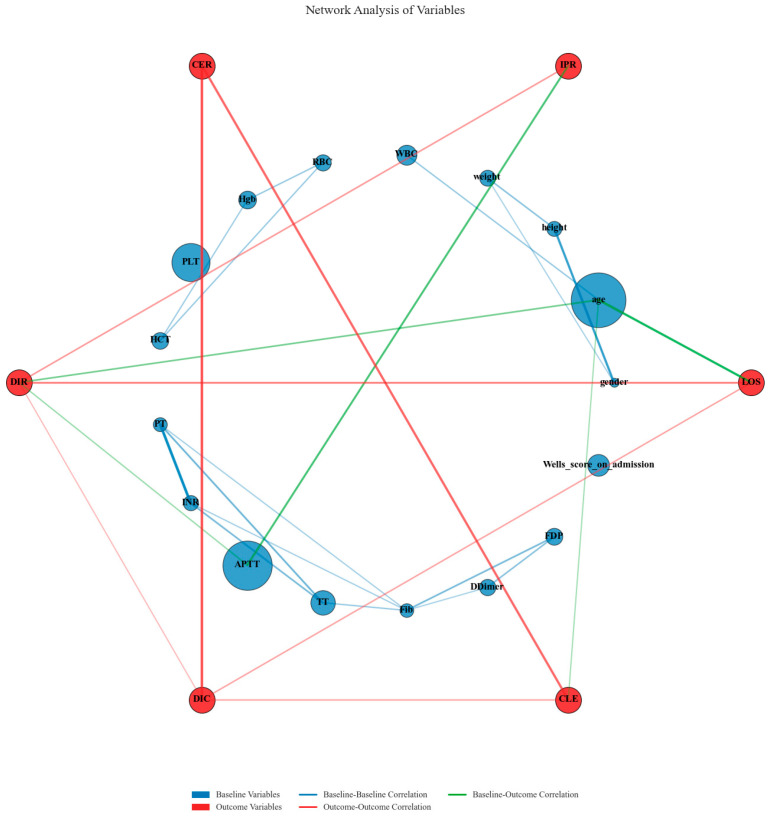
Network analysis of relationships between baseline variables and clinical outcomes.

Network analysis of variables revealed intricate relationships between baseline clinical parameters and outcome measures across multiple matching datasets. The visualization demonstrated strong correlations (correlation coefficient >0.3) between several baseline coagulation indicators and treatment outcomes. APTT and PLT exhibited substantial node sizes, indicating their considerable weighted importance in predicting outcomes. Strong baseline–baseline correlations were observed between coagulation markers, particularly within the APTT-TT-Fib-Dimer cluster and the PLT-WBC-Hgb-HCT cluster, reflecting the integrated nature of the coagulation system. Critical outcome–outcome correlations were identified between DIC and DIR, and between CLE and LOS, demonstrating the interconnected nature of cost efficiency and clinical improvement metrics. Notable baseline–outcome correlations were observed between APTT and DIC, and between PLT and IPR, suggesting these baseline coagulation parameters serve as critical mediators of treatment response. This network analysis complements the previous sensitivity assessment by illuminating the mechanistic pathways through which baseline coagulation profiles influence clinical and economic outcomes, thereby providing a biological foundation for understanding the observed treatment effects between CAV and SAT groups (Figure 12).

## 4. Discussion

This retrospective cohort study demonstrates that CAV with rivaroxaban and Aescuven offers significant advantages over SAT with rivaroxaban alone in DVT management. Through rigorous propensity score matching and counterfactual analysis, we established causal relationships between treatment modalities and multidimensional outcomes. The CAV regimen exhibited superior clinical efficacy with a 6.39 percentage point increase in improvement rate, enhanced economic efficiency with a 3323.38 unit reduction in daily improvement cost, and had a minimal impact on healthcare resource utilization with only a marginal 0.15-day increase in length of stay.

Standard DVT treatment relies on anticoagulants including rivaroxaban, which prevents thrombus progression and reduces complications [13]. Additionally, medical interventions such as catheter-directed thrombolysis and mechanical thrombectomy are utilized in certain cases for direct clot removal. Beyond these direct treatments targeting the thrombus itself, Aescuven, as a plant-derived complementary therapy, is a medication based on horse chestnut (Aesculus hippocastanum) seed extract, with aescin as its primary active component [14]. Aescin exerts anti-inflammatory effects by inhibiting cyclooxygenase and lipoxygenase, thereby reducing pro-inflammatory mediators [15,16]. Additionally, aescin demonstrates anti-edema properties through hyaluronidase inhibition [17,18]. By preserving hyaluronic acid integrity, aescin maintains vascular barrier function, reduces permeability, and alleviates DVT-related edema [19,20]. Inflammation in DVT pathophysiology activates additional coagulation pathways through inflammatory mediators like IL-1β and TNF-α, potentially reducing anticoagulation efficacy [21,22]. The impact of edema on DVT treatment is primarily manifested in skin changes due to persistent edema, leading to complications such as cellulitis and venous ulcers, which undoubtedly delay patient recovery and extend medication duration [23,24]. Edema also impairs anticoagulant distribution, potentially requiring higher dosages, increasing costs and bleeding risks [25,26,27]. Our counterfactual analysis confirms that incorporating Aescuven into DVT treatment enhances therapeutic outcomes by supporting venous health, achieving dual clinical and economic benefits without significantly extending hospitalization.

Notably, our network analysis revealed a complex association mechanism between baseline coagulation parameters and treatment efficacy. The significant correlation between APTT, an indicator of the intrinsic coagulation pathway, and the economic benefit indicator DIC, demonstrates a direct link between coagulation system activation level and the economic advantages of the CAV regimen. This finding not only explains the observed heterogeneity in treatment effects but also provides a theoretical foundation for precision medicine approaches in clinical practice. The APTT–economic benefit relationship aligns with Aescuven’s mechanism of inhibiting inflammatory mediators and reducing tissue factor expression [28,29]. More importantly, this systematic association between baseline coagulation parameters and treatment response reveals potential biomarkers for individualizing DVT therapy, as patients with varying degrees of coagulation dysfunction respond differently to the CAV regimen [30,31]. Pathophysiologically, these findings support Aescuven’s dual role in inflammation suppression and endothelial protection, creating a favorable environment for anticoagulation [32]. This mechanistic understanding transcends the simple description of clinical phenomena, providing mechanism-based theoretical support for developing comprehensive strategies for DVT management.

Another important dimension of our economic analysis is the temporal efficiency of resource utilization, with the CAV regimen demonstrating a remarkable cost–time asymmetry effect. While hospitalization duration remained virtually unchanged, the cost per unit of clinical improvement decreased substantially, creating a “time compression effect” across all matching methods [33,34,35]. This effect represents a qualitative transformation in resource utilization efficiency rather than simple cost reduction. More notably, the magnitude of decrease in clinical improvement costs far exceeded the linear combination of increased improvement rate and marginally extended hospital stay, revealing the synergistic effect of the CAV regimen [36]. This non-linear benefit pattern likely stems from Aescuven’s ability to suppress inflammation and protect vascular endothelium, creating a more favorable pharmacological environment for rivaroxaban, thus achieving a “1 + 1 > 2” therapeutic effect [37]. From a hospital management perspective, the significant improvement in health output per bed-day means that the same medical resources can generate greater health value [38,39]. For every hundred DVT patients on the CAV regimen, an addition of just 15 hospital days saves costs equivalent to treating 33 patients, offering valuable evidence for resource allocation in healthcare systems.

This study has several limitations that should be considered when interpreting its findings. First, as a secondary analysis of an existing clinical cohort using propensity score matching methods, we cannot completely eliminate selection bias despite employing a multi-method matching framework and ensemble weighting approach. While these sophisticated techniques strengthen causal inference, they cannot fully account for unmeasured confounders that may have influenced treatment allocation. Second, the retrospective, single-center design inherently introduces regional constraints and institutional biases, potentially limiting the generalizability of our findings to other healthcare settings with different clinical practices, patient populations, and treatment protocols. Third, our outcome assessment was confined to the inpatient period until discharge, precluding evaluation of long-term benefits and potential delayed effects of the CAV regimen. This limitation is particularly relevant considering that DVT-related complications such as post-thrombotic syndrome typically manifest over extended timeframes. Future research would benefit from prospective, multicenter randomized controlled trials with prolonged follow-up periods to validate our findings and assess long-term clinical outcomes, recurrence rates, and persistent economic advantages of combining venoprotective agents with standard anticoagulation therapy.

## 5. Conclusions

This study presents a comprehensive evaluation framework for assessing the multifaceted benefits of combining venoprotective therapy with standard anticoagulation in DVT management. Through rigorous causal inference methodology, we demonstrated that the CAV regimen offers significant advantages across clinical, economic, and resource utilization dimensions compared to standard rivaroxaban monotherapy. The integration of vascular protection principles into conventional anticoagulation strategies represents a paradigm shift from single-target to multi-target therapeutic approaches, addressing not only thrombosis itself but also its inflammatory and vascular pathophysiological components. By simultaneously improving clinical outcomes and enhancing economic efficiency without significantly increasing resource utilization, the CAV regimen exemplifies a value-based healthcare approach that optimizes both patient and system-level benefits. These findings underscore the importance of holistic treatment frameworks that consider the complex interplay between coagulation, inflammation, and vascular integrity in DVT pathophysiology. Beyond specific pharmacological interventions, this research establishes the value of comprehensive assessment methodologies that capture the multidimensional nature of therapeutic benefits, providing a model for evaluating complex interventions in cardiovascular medicine and informing evidence-based strategies for DVT management.

## Figures and Tables

**Table 1:** Baseline characteristics of patients after different matching methods.

	Genetic Matching				Mahalanobis Distance Matching				Nearest Neighbor Caliper Matching				Optimal Exact Matching			
Characteristic	N	SAT (N = 48)	CAV (N = 48)	*p*-Value	N	SAT (N = 48)	CAV (N = 48)	*p*-Value	N	SAT (N = 48)	CAV (N = 48)	*p*-Value	N	SAT (N = 48)	CAV (N = 48)	*p*-Value
Gender	96			0.15	96			0.066	68			0.81	96			>0.99
Female		26 (54%)	19 (40%)			28 (58%)	19 (40%)			15 (44%)	16 (47%)			19 (40%)	19 (40%)	
Male		22 (46%)	29 (60%)			20 (42%)	29 (60%)			19 (56%)	18 (53%)			29 (60%)	29 (60%)	
Age	96	68 (53, 76)	63 (49, 74)	0.27	96	66 (52, 77)	63 (49, 74)	0.23	68	64 (51, 74)	63 (52, 75)	0.72	96	64 (50, 74)	63 (49, 74)	0.54
Height	96	159 (156, 163)	162 (157, 170)	0.1	96	158 (156, 162)	162 (157, 170)	0.037	68	160 (157, 165)	160 (156, 167)	0.86	96	160 (158, 166)	162 (157, 170)	0.54
Weight	96	60 (57, 66)	63 (57, 70)	0.32	96	60 (55, 65)	63 (57, 70)	0.053	68	60 (57, 66)	60 (56, 67)	0.97	96	61 (56, 66)	63 (57, 70)	0.32
WBC	96	9.75 (8.40, 11.30)	9.25 (8.17, 10.60)	0.066	96	10.21 (8.40, 11.58)	9.25 (8.17, 10.60)	0.034	68	9.45 (8.35, 11.18)	9.94 (8.88, 10.69)	0.83	96	9.95 (8.42, 11.56)	9.25 (8.17, 10.60)	0.038
RBC	96	5.17 (4.92, 5.43)	5.01 (4.75, 5.28)	0.12	96	5.04 (4.83, 5.29)	5.01 (4.75, 5.28)	0.49	68	5.01 (4.75, 5.30)	4.99 (4.77, 5.23)	0.66	96	5.01 (4.79, 5.38)	5.01 (4.75, 5.28)	0.7
Hgb	96	14.77 (14.15, 15.69)	15.13 (14.67, 15.50)	0.17	96	14.85 (14.30, 15.62)	15.13 (14.67, 15.50)	0.26	68	14.85 (14.31, 15.77)	15.15 (14.52, 15.47)	0.64	96	15.08 (14.34, 15.75)	15.13 (14.67, 15.50)	0.65
PLT	96	154 (119, 203)	146 (116, 168)	0.29	96	160 (129, 201)	146 (116, 168)	0.091	68	156 (119, 193)	147 (126, 189)	0.76	96	158 (122, 203)	146 (116, 168)	0.12
HCT	96	45.23 (44.57, 45.96)	45.55 (44.43, 46.29)	0.91	96	45.64 (44.64, 45.98)	45.55 (44.43, 46.29)	0.78	68	45.53 (44.52, 45.97)	45.39 (43.99, 46.06)	0.79	96	45.61 (44.57, 46.08)	45.55 (44.43, 46.29)	0.87
PT	96	13.35 (12.07, 14.53)	13.70 (12.47, 15.03)	0.36	96	13.45 (11.75, 14.50)	13.70 (12.47, 15.03)	0.28	68	13.70 (12.45, 14.57)	13.70 (12.55, 15.15)	0.76	96	13.70 (12.38, 15.22)	13.70 (12.47, 15.03)	>0.99
INR	96	1.11 (0.94, 1.19)	1.12 (1.01, 1.25)	0.45	96	1.11 (0.93, 1.19)	1.12 (1.01, 1.25)	0.39	68	1.14 (1.05, 1.22)	1.12 (1.01, 1.26)	0.86	96	1.12 (1.00, 1.23)	1.12 (1.01, 1.25)	0.88
APTT	96	32.4 (26.9, 36.3)	28.9 (26.4, 32.5)	0.13	96	31.6 (27.6, 36.3)	28.9 (26.4, 32.5)	0.095	68	29.4 (27.0, 34.5)	29.1 (26.4, 34.1)	0.83	96	31.4 (27.1, 35.2)	28.9 (26.4, 32.5)	0.24
TT	96	24.8 (21.3, 29.9)	27.0 (24.7, 30.4)	0.082	96	24.8 (21.2, 28.8)	27.0 (24.7, 30.4)	0.028	68	27.9 (24.0, 30.3)	26.8 (24.1, 29.4)	0.72	96	27.9 (24.2, 30.2)	27.0 (24.7, 30.4)	0.97
Fib	96	3.56 (3.04, 4.10)	3.45 (2.88, 3.88)	0.3	96	3.66 (3.06, 4.05)	3.45 (2.88, 3.88)	0.15	68	3.58 (3.07, 3.88)	3.41 (2.96, 3.88)	0.71	96	3.48 (3.01, 3.84)	3.45 (2.88, 3.88)	0.91
DDimer	96	11.2 (7.9, 14.5)	11.0 (7.8, 16.2)	0.96	96	11.4 (8.3, 14.8)	11.0 (7.8, 16.2)	0.83	68	11.7 (7.9, 14.4)	10.6 (7.9, 14.6)	0.98	96	12.1 (8.2, 15.3)	11.0 (7.8, 16.2)	0.69
FDP	96	19 (13, 25)	21 (15, 27)	0.5	96	20 (14, 24)	21 (15, 27)	0.51	68	21 (12, 24)	20 (13, 26)	0.63	96	20 (12, 26)	21 (15, 27)	0.6
Wells score on admission	96	4.21 (3.18, 4.50)	3.89 (3.70, 4.25)	0.69	96	4.33 (3.18, 4.50)	3.89 (3.70, 4.25)	0.46	68	4.36 (3.54, 4.50)	4.00 (3.82, 4.49)	>0.99	96	4.44 (3.55, 4.50)	3.89 (3.70, 4.25)	0.15

**Table 2:** Treatment effect analysis across multiple outcomes and matching methods.

Outcome	Method	ATE	ATT	ATC	DR Effect
LOS	Genetic matching	0.220938	0.138542	0.303333	0.234141
	Mahalanobis matching	0.030104	0.069583	−0.009375	0.060673
	Nearest caliper matching	0.184853	0.140294	0.229412	0.482037
	Optimal exact matching	0.122396	0.093542	0.151250	−0.089078
	Weighted average	0.149940	0.115998	0.183882	0.215417
IPR	Genetic matching	7.250269	7.126380	7.374158	6.657812
	Mahalanobis matching	9.498363	10.104076	8.892649	7.468033
	Nearest caliper matching	4.425388	4.053835	4.796941	4.576757
	Optimal exact matching	6.234179	5.597047	6.871312	4.811417
	Weighted average	6.390419	6.185866	6.594972	5.609704
CER	Genetic matching	376.835863	331.227682	422.444043	49.228880
	Mahalanobis matching	234.975301	219.382172	250.568430	−31.334780
	Nearest caliper matching	−382.278629	−558.997681	−205.559576	17.847259
	Optimal exact matching	443.893589	152.772527	735.014650	204.663569
	Weighted average	92.494279	−53.786728	238.775285	60.460884
DIR	Genetic matching	1.126066	1.237232	1.014900	0.081863
	Mahalanobis matching	1.040437	0.868627	1.212247	0.054384
	Nearest caliper matching	−0.510985	−0.746027	−0.275944	−0.546280
	Optimal exact matching	0.854262	0.735216	0.973309	0.295889
	Weighted average	0.453157	0.333016	0.573299	−0.099355
DIC	Genetic matching	−2223.558387	−3261.261570	−1185.855203	−2897.334079
	Mahalanobis matching	−2687.825196	−3521.714681	−1853.935712	−3658.028076
	Nearest caliper matching	−4575.084249	−5472.104670	−3678.063828	−2258.112576
	Optimal exact matching	−2914.259329	−3844.182311	−1984.336347	−3252.662104
	Weighted average	−3323.379356	−4247.677021	−2399.081692	−2887.947738
CLE	Genetic matching	146.768519	121.704870	171.832168	144.049049
	Mahalanobis matching	145.561282	123.163489	167.959075	159.493708
	Nearest caliper matching	83.567817	92.234486	74.901147	117.317895
	Optimal exact matching	202.467521	167.803752	237.131289	189.321178
	Weighted average	136.974290	122.308297	151.640284	147.968124

## Data Availability

The dataset provided in this paper is not easily accessible, as these data are part of an ongoing study. Requests for access to the dataset should be sent directly to ymlim@utar.edu.my. The complete source code used for all analyses in this study, including propensity score matching, ensemble weighting, and counterfactual analyses, is freely available at https://github.com/ZhouNan2020/ACV_RV (accessed on 11 April 2025). This repository contains all necessary scripts and documentation for reproducing our analyses.

## Supplementary Materials

The following supporting information can be downloaded at: https://www.mdpi.com/article/10.3390/reports8020083/s1, Table S1: Baseline comparison of patients in the original cohort.

## Abbreviations

The following abbreviations are used in this manuscript:

DVT	deep vein thrombosis
CAV	combined anticoagulation and venoprotective therapy
SAT	standard anticoagulation therapy
IPR	improvement rate
DIR	daily improvement rate
CER	cost-effectiveness ratio
DIC	daily improvement cost
CLE	cost–LOS efficiency
LOS	length of stay
SMD	standardized mean difference
ATE	average treatment effect
ATT	average treatment effect on the treated
ATC	average treatment effect on controls
ITE	individual treatment effect
APTT	activated partial thromboplastin time
PT	prothrombin time
INR	international normalized ratio
TT	thrombin time
Fib	fibrinogen
FDP	fibrin degradation product
WBC	white blood cell
RBC	red blood cell
Hgb	hemoglobin
PLT	platelet
HCT	hematocrit
VTE	venous thromboembolism
PE	pulmonary embolism
DOACs	direct oral anticoagulants
VKA	vitamin K antagonist
CVI	chronic venous insufficiency
PTS	post-thrombotic syndrome
NOAC	novel oral anticoagulant
PPPM	per patient per month
COX	cyclooxygenase
IL	interleukin
TNF-α	Tumor Necrosis Factor-alpha
ICAM-1	Intercellular Adhesion Molecule-1
CEC	circulating endothelial cell
ET-1	Endothelin-1
sTM	soluble thrombomodulin
PaO2	arterial partial pressure of oxygen
QALY	quality-adjusted life year
PST	patient self-testing
TTR	time in therapeutic range
PSA	probabilistic sensitivity analysis
SDV	source data verification
GCP	good clinical practice
