# Supplementary material for: Multidimensional Evaluation of Combined Anticoagulation and Venoprotective Therapy in Deep Vein Thrombosis: A Retrospective Propensity Score-Matched Cohort Study of Clinical, Economic, and Resource Utilization Outcomes
Source: Reports (MDPI). 2025 Jun 1;8(2):83. doi: 10.3390/reports8020083 (PMC12197247; doi:10.3390/reports8020083)
Supplement: Supplementary file 1 [file reports-08-00083-s001.zip › reports-3611934-supplementary.pdf]

Supplementary File Table S1 Baseline comparison of patients in the original cohort

Characteristic	N	SAT (N = 348)	CAV（N = 48）	p-value
Gender	396			0.035
Female		194 (56%)	19 (40%)	
Male		154 (44%)	29 (60%)	
Age	396	63 (52, 73)	63 (49, 74)	0.75
Height	396	160 (156, 167)	162 (157, 170)	0.27
Weight	396	60 (54, 66)	63 (57, 70)	0.063
Wbc	396	9.95 (8.58, 11.51)	9.25 (8.17, 10.60)	0.009
Rbc	396	5.03 (4.75, 5.33)	5.01 (4.75, 5.28)	>0.99
Hgb	396	15.02 (14.14, 15.80)	15.13 (14.67, 15.50)	0.43
Plt	396	158 (124, 208)	146 (116, 168)	0.048
Hct	396	45.15 (44.14, 46.06)	45.55 (44.43, 46.29)	0.33
Pt	396	13.00 (11.80, 14.40)	13.70 (12.47, 15.03)	0.063
Inr	396	1.06 (0.95, 1.19)	1.12 (1.01, 1.25)	0.064
Aptt	396	33.5 (30.0, 36.7)	28.9 (26.4, 32.5)	<0.001
Tt	396	24.8 (21.5, 28.9)	27.0 (24.7, 30.4)	0.003
Fib	396	3.46 (2.88, 4.09)	3.45 (2.88, 3.88)	0.44
Ddimer	396	11.9 (9.0, 15.5)	11.0 (7.8, 16.2)	0.37
Fdp	396	18 (12, 25)	21 (15, 27)	0.14
Wells_Score_On_Admission	396	4.36 (3.00, 4.50)	3.89 (3.70, 4.25)	0.98
